# Petting Zoo Animals as an Emerging Reservoir of Extended-Spectrum β-Lactamase and AmpC-Producing Enterobacteriaceae

**DOI:** 10.3389/fmicb.2019.02488

**Published:** 2019-10-30

**Authors:** Anat Shnaiderman-Torban, Amir Steinman, Gal Meidan, Yossi Paitan, Wiessam Abu Ahmad, Shiri Navon-Venezia

**Affiliations:** ^1^Koret School of Veterinary Medicine, The Robert H. Smith Faculty of Agriculture, Food and Environment, The Hebrew University of Jerusalem, Rehovot, Israel; ^2^Department of Clinical Microbiology and Immunology, Sackler Faculty of Medicine, Tel Aviv University, Tel Aviv, Israel; ^3^Clinical Microbiology Lab, Meir Medical Center, Kfar Saba, Israel; ^4^Hebrew University-Hadassah Braun School of Public Health and Community Medicine, Jerusalem, Israel; ^5^Department of Molecular Biology, Faculty of Natural Sciences and Adelson School of Medicine, Ariel University, Ariel, Israel

**Keywords:** petting zoos, animals, ESBL, AmpC, environmental shedding, Enterobacteriaceae, risk factors

## Abstract

Extended spectrum beta-lactamases and AmpC-producing Enterobacteriaceae (ESBL/AmpC-E) have become a great concern in both human and veterinary medicine. One setting in which this risk could be particularly prominent is petting zoos, in which humans, especially children, directly and indirectly interact with the animals. Yet, while the zoonotic transmission of various Enterobacteriaceae has been reported previously in petting zoos, reports on ESBL/AmpC-E shedding in this setting is currently lacking, despite the high potential risk. To fill this knowledge gap, we conducted a prospective cross-sectional study to explore the prevalence, molecular epidemiology, and risk for shedding of ESBL/AmpC-E in petting zoos. We performed a prospective cross-sectional study in eight petting zoos. Altogether, we collected 381 fecal and body-surface samples from 228 animals, broth-enriched them, and then plated them onto CHROMagar ESBL-plates for ESBL/AmpC-E isolation. Next, we identified the isolated species and tested their susceptibility to various antibiotics using the Vitek-2 system, determined bacterial relatedness by multilocus sequence typing (MLST), and identified ESBL/AmpC genes by using PCR and sequencing. Finally, we asked petting zoo owners and veterinarians to complete questionnaires, which we then analyzed to evaluate risk factors for ESBL/AmpC-E shedding. We found that ESBL/AmpC-E shedding is an important, currently oversighted risk in petting zoos, as the overall shedding rate was 12% (35 isolates, including 29% ESBL-producers, 34% AmpC-producers, and 37% ESBL and AmpC-producers). The isolated bacteria included *Enterobacter cloacae* (55%), *Escherichia coli* (31%), and *Citrobacter freundii* (14%), with diverse ESBL genes. MLST revealed diverse sequence types (STs), including the highly virulent Enterotoxigenic ST656 and the Uropathogenic ST127 *E. coli* strains, indicating complex epidemiology with inter-animal bacterial transmission. Shedding was associated with petting permission and antibiotic treatment in the petting zoo (OR = 7.34), which were identified as risk factors for ESBL/AmpC shedding. Our findings highlight petting zoos as a source for antibiotic-resistant ESBL/AmpC-producing bacteria, including highly virulent, disease-associated MDR *E. coli* strains. As this risk has not been previously described in detail, it calls for the implementation of infection control and active surveillance programs in petting zoos and raises the need for a comprehensive guideline to restrain this emerging concern.

## Introduction

Petting zoos – either permanent or temporary – are popular attraction sites that allow both direct and indirect exposure of children and adults to diverse animals (Steinmuller et al., 2006). Despite the educational and entertainment value of such interactions, petting zoos raise a significant concern regarding the zoonotic transmission of pathogens due to contact with animals, mainly through the oral–fecal route (Conrad et al., 2016). Indeed, the US Centers for Disease Control and Prevention (CDC) has published recommendations on “how to stay healthy at animal exhibits”^1^. Previous reports of public health risks and zoonosis originating from petting zoos, mostly in North America, typically describe either the transmission of highly virulent bacterial pathogens, including *Escherichia coli* and *Salmonella* outbreaks in petting zoos (Friedman et al., 1998; Goode et al., 2009), or risk behaviors for disease transmission (Weese et al., 2007; Erdozain et al., 2013).

In the past two decades, the global incidence of plasmid-mediated AmpC and extended spectrum beta-lactamases (ESBL)-producing Enterobacteriaceae (ESBL/AmpC-E) has increased constantly in both humans and animals (Schwaber et al., 2006; Barco et al., 2015; Leonard et al., 2015). Environmental shedding of ESBL/AmpC-E by farm animals, such as cattle, poultry, and swine has been widely investigated in the past (Horton et al., 2011), but it is alarmingly understudied in petting zoos. In a single study, ESBL-producing *E. coli* were isolated from feces of petting zoo animals (Conrad et al., 2018), but larger studies on the incidence and risk factors for ESBL/AmpC-E shedding in petting zoos are lacking. Due to the direct contact between visitors (mainly children) and animals, identifying and characterizing the presence of these antibiotic-resistant bacteria on the body surface of the animals and the possible environmental shedding is of great public importance. Accordingly, in this prospective study, our aim was to determine the prevalence of ESBL/AmpC-E shedding in various petting-zoo animal species, characterize the molecular epidemiology of the isolates, and define the risk factors for shedding.

Addressing the emerging threat of antibiotic-resistant bacteria in petting zoo animals requires a “One Health” perspective and, therefore, the data from this study are crucial to the fight against the spread of resistance.

## Materials and Methods

### Petting Zoos and Study Design

We conducted a prospective cross-sectional study in eight permanent petting zoos across Israel (December 2016–May 2017), chosen randomly. The study was approved by the Internal Ethics Committee of the Koret School of Veterinary Medicine, Israel (Protocol KSVM-VTH/25_2016), and was made possible through a respectable collaboration with the facility owners and veterinarians. We recruited petting zoos based on owners’ cooperation, considering the appropriate sample size. In order to examine diverse risk factors for shedding, we chose petting zoos which differed in characteristics (schools, exhibition, in a zoo property and ambulatory). In interviews conducted with the owners, we collected demographic and medical data, throughout owners’ questioners. Data included the total number of animals in each facility, the number of animal species, the type of veterinary care, petting and feeding policies by visitors and employees, the number of employees, and the average daily number of visitors. Data on each sampled animal included its class, species, diet, and sex, and antibiotic treatments that had been received during the past year, according to owners’ questioner.

### Bacteria Sampling, Isolation, Identification, and Antibiotic Susceptibility Testing

In each petting zoo, we sampled the maximum number of animals from diverse species. The goal was to sample all animals housed in the petting zoo, whereas in practice we sampled the animals which the owners approved to sample, mainly due to safety considerations. Sampling was performed during morning-noon hours. We collected fecal specimens from the close vicinity of the animal and analyzed them only if we could link them, through direct observation, to a specific animal. According to the decision of the owners and the ability to safely approach the animal, we also collected surface specimens from the skin, fur, or feathers by rubbing a sterile cotton swab, pre-moistened with saline, on the surface of the animal, in the back area, for at least 10 s. The sampling area was proportional to the size of each sampled animal.

Samples were stored at room temperature, in the commercial transport gel and were processed within 24 h of sampling. All samples were inoculated into 2-mL of a brain–heart infusion enrichment broth, so as to increase the sensitivity of ESBL and AmpC-E detection (Murk et al., 2009). After incubation of 18–24 h at 37°C, the enriched cultures were plated (10 μL) onto Chromagar ESBL plates (Hy-Labs, Rehovot, Israel). Colonies that appeared after an overnight incubation at 37°C were recorded, and one colony of each distinct color was re-streaked onto a fresh Chromagar ESBL plate to obtain a pure culture. Next, the pure ESBL/AmpC-E suspected isolates were stored at −80°C stocks for further workup. All isolates were subjected to Vitek-2 for species identification and antibiotic susceptibility testing (AST-N270 Vitek2 card, BioMérieux, Inc., Marcy-l’Etoile, France). The identification of *Enterobacter* and *Citrobacter* species was verified using 16S rRNA gene sequencing and an RDP database comparison (Cole et al., 2005). ESBL and AmpC production were confirmed using combination disc diffusion confirmatory assays and interpreted according to the CLSI guidelines (CLSI, 2017, 27th edition). We adopted the guidelines for *E. coli* ESBL confirmatory assay by disc diffusion, and implemented it for *Enterobacter cloacae* and for *Citrobacter freundii*. All *E. cloacae* and for *C. freundii isolates* were automatically defined as AmpC producers, due to intrinsic resistance. Plasmid mediated AmpC production was tested via cefoxitin and resistance to second generation cephalosporin.

### Genotyping and Detection of β-Lactamase Genes of ESBL/AmpC-E

To determine the genetic relatedness between isolates belonging to the same species, genotyping was performed using an Enterobacterial repetitive intergenic consensus (ERIC) PCR amplification with the following primer: 5′-AAG TAAGTGACTGGGGTGAGCG-3′ (Versalovic et al., 1991). Results were analyzed using GelJ software (Heras et al., 2015) and all strains exhibiting a distinct ERIC PCR pattern were subjected to multilocus sequence type (MLST) using schemes for *E. coli*, *E. cloacae*, and *C. freundii*, as described previously (Diancourt et al., 2005; Bai et al., 2012; Miyoshi-Akiyama et al., 2013). Sequences of new gene alleles and sequence types (STs) were submitted to and assigned by PubMLST^2^.

Extended spectrum beta-lactamases and AmpC β-lactamase genes were identified by PCRs and sequencing. Isolates were examined for the presence of *bla*_CMY–__1_, *bla*_CMY–__2_ (Kim et al., 2005), *bla*_CTX–M_ group (Woodford et al., 2006), *bla*_OXA–__1_, *bla*_OXA–__2_, *bla*_OXA–__10_ (Lin et al., 2012), *bla*_TEM_, and *bla*_SHV_ groups (Tofteland et al., 2007). Genes identified as *bla*_CTX–M–__1_ and *bla*_CTX–M–__9_ groups were sequenced to identify the specific gene allele, using the following primers (designed in this study): *bla*_CTX–M–__1_F- ATGGTTAAAAAATCACTGCG and *bla*_CTX–M–__1_R TTACAAACCGTTGGTGACG, *bla*_CTX–M–__9_F- ATGGTGACAAAGAGAGTGCAAC and *bla*_CTX–M–__9_R TTACAGCCCTTCGGCGATGA, respectively.

### Statistical and Risk Factor Analyses

The minimal sample size (number of animals sampled) was calculated using WinPepi, based on an estimated shedding rate of 10% for ESBL-E in community companion animals in Israel (A. Shnaiderman-Torban, unpublished), with a confidence level of 95% and an acceptable difference of 5%, resulting in *n* = 139. Statistical analyses were performed using the IBM STATISTICS SPSS software (SPSS Version 24; SPSS Inc., Chicago, IL, United States). Data distribution was examined by testing whether the Skewness and Kurtosis equal zero and by performing the Shapiro–Wilk’s test. Continuous variables were analyzed using *t*-tests or Mann–Whitney *U*-tests, according to the distribution of the variable. Categorical variables were analyzed using the Fisher’s exact test or the Pearson chi-square test, as appropriate. In all statistical analyses, *p* ≤ 0.05 indicated significance. A multiple logistic regression model, using the ENTER method, was applied for ESBL/AmpC-E shedding using variables with *p* ≤ 0.2.

## Results

### Characterization of Petting Zoos and Animal Populations

The study population included animals in eight petting zoos in Israel, which were diverse in type, size, and other characteristics (Table 1). Overall, 228 animals (42 species) were sampled for ESBL/AmpC gut-shedding, including 161 mammals (71%, 23 species), 47 reptiles (20%, 12 species), and 20 avian species (9%, 7 species). Altogether, 381 specimens were collected from these animals, including fecal samples from 52 animals, surface samples (skin/fur/feathers) from 23 animals, and both fecal and surface samples from153 animals.

**TABLE 1:** Characteristics of the eight petting zoos included in this study.

**Petting zoo**	**Type of**	**per zoo**	**No. of animal**	**No. of**	**Average daily**	**Permitted**		**No. of animals treated with**
	**facility**	**per zoo**	**species**	**employees**	**visitors**	**policy**		**antibiotics at sampling (%)^a^**
						**Petting**	**Eating**	
1	Zoo property	<50	15	6	50	+	−	0
2	Exhibition^b^		20	3	50	−	+	8/19 (42)
3	Zoo property		10	8	150	+	−	0
4	Ambulatory^c^	50–100	30	2	10	+	−	4/24 (17)
5	School property		20	3	50	+	+	2/46 (4)
6	School property		30	2	10	+	−	2/53 (4)
7	Private	>100	20	9	70	+	+	0
8	Zoo property		35	14	>100	+	−	5/38 (13)

*^*a*^Percent of antibiotic-treated animals at the sampling day, out of the total sampled animals at the respective petting zoo. ^*b*^Restricted only for exhibitions with a petting prohibition policy. ^*c*^Used for celebrations of children parties and festivals.*

### Prevalence of ESBL and/AmpC-Producing Enterobacteriaceae (ESBL/AmpC-E)

**FIGURE 1:**
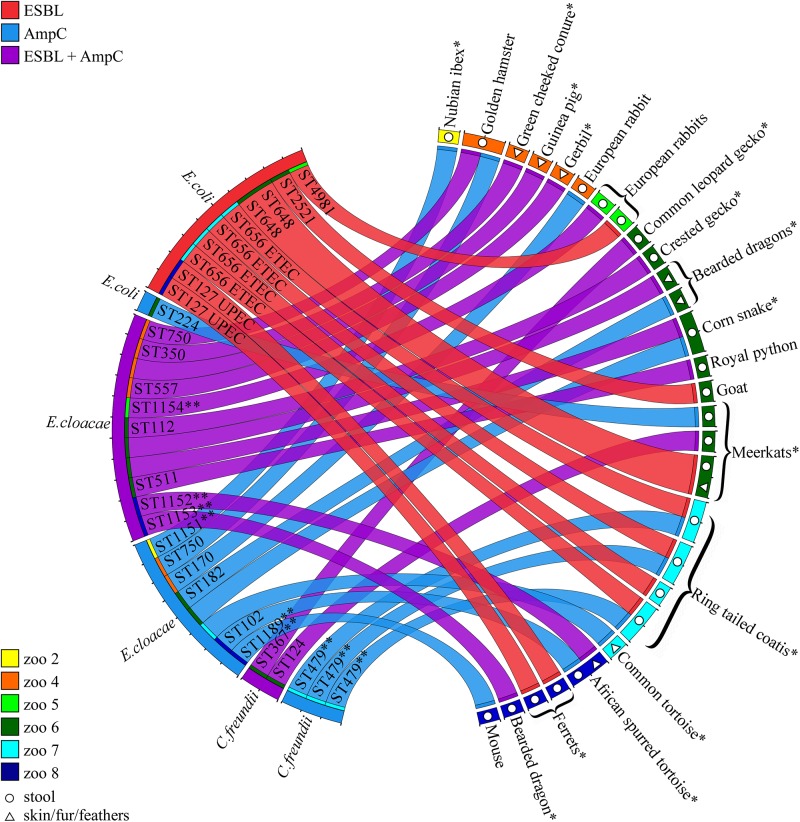
ESBL/AmpC-E isolates recovered from petting zoo animals. Circos diagram presenting the 35 ESBL/AmpC-E isolates (left) recovered from 28 animals and their housing petting zoos (right; each animal is represented by a colored square). The bacterial species, sequence type (ST), resistance phenotype (ESBL, AmpC, or both; represented by the color of the ribbon), and isolation source [feces, represented by open circles, or surface (skin/fur/feathers), represented by open triangles] are designated for each isolate. Animal species described here for the first time as shedding ESBL/AmpC-E are designated with an ^∗^. New bacterial STs are designated with ^∗∗^. ESBL/AmpC-E isolates that are represented by a ribbon with an unassigned ST represent bacteria that were not preserved for further investigation due to technical reasons. UPEC, Uropathogenic *E. coli*; ETEC, Enterotoxigenic *E. coli*.

Of the 228 sampled animals, 12% (*n* = 28, CI 95% 8–17%) carried at least one strain of ESBL/AmpC-E and 25% co-carried more than one antibiotic-resistant strain (Figure 1, Table 2, and Supplementary Table S1). Carriage rates within different petting zoos varied significantly, from 0 to 22% (Table 2). Overall, 35 ESBL/AmpC-E isolates were recovered from 28 animals, of which 77% were from feces samples and 23% were from surface samples (*n* = 27 and eight samples, respectively; Figure 1, Table 2, and Supplementary Table S1).

**TABLE 2:** Shedding rates of ESBL and AmpC-producing *Enterobacteriaceae* in petting zoos.

**Petting zoo**	**No. of sampled animals**	**Number of positive ESBL/AmpC shedding animals (%) and bacterial species**			**Total No. of ESBL/AmpC carriers (%)**
		**Mammals**	**Reptiles**	**Avian species**	
1	10	0/10 (0)	Not sampled	Not sampled	0
2	19	1/14 (7) *E. cloacae complex* (1)	0/2 (0)	0/3 (0)	1/19(5)
3	15	0/15 (0)	Not sampled	Not sampled	0
4	24	4/17 (24)^a^ *E. cloacae complex* (5)	0/4 (0)	1/3 (30) *E. cloacae complex* (1)	5/24(20)
5	46	2/33 (6) *E. cloacae complex* (1); *E. coli* (1)	0/5 (0)	0/8 (0)	2/46(4)
6	53	4/25 (16)^a^ *C. freundii* (1); *E. coli* (4)	6/23 (30)^a^ *E. cloacae complex* (6); *C. freundii* (1)	0/5 (0)	10/53(19)
7	23	4/16 (25)^b^ *C. freundii* (3); *E. coli* (4)	1/6 (17) *E. cloacae complex* (1)	0/1 (0)	5/23(22)
8	38	3/31 (10) *E. cloacae complex* (1); *E. coli* (2)	2/7 (29) *E. cloacae complex* (3)	Not sampled	5/38(13)
Total	228	18/161 (11)	9/47 (19)	1/20 (5)	28/228(12)

*^*a*^One animal shed two bacterial species. ^*b*^Three animals shed two bacterial species each, and one animal shed one bacterial species.*

Of the 153 animals that were sampled from both feces and body surface, 15 animals were positive for ESBL/AmpC-E only in fecal samples, four animals were positive only in surface samples, and two animals were positive in both fecal and surface samples: a turtle from petting zoo #8, which carried two different *E. cloacae* strains on the skin (ST102) and in the feces (ST1152), with different resistance phenotypes (isolates p151.2 and p152.2; Supplementary Table S1); and a meerkat from petting zoo #6, which carried the same ESBL-producing *E. coli* strain, ST648, on its fur and in its feces (isolates p381.2 and p382.2; Supplementary Table S1).

Of the 42 animal species that were sampled, 19 species carried ESBL/AmpC-E (11 mammals, 7 reptiles, and one avian species). To the best of our knowledge, for 13 of these animal species, this is the first report describing ESBL/AmpC-E shedding (Figure 1 and Supplementary Table S1).

More than half (55%, *n* = 19) of the 35 ESBL/AmpC-producing isolates belonged to the genera *E. cloacae* complex, while the rest were identified as *E. coli* (31%, *n* = 11) and *C. freundii* (14%, *n* = 5). The isolates encompassed strains that produce both ESBLs and AmpC (37%, *n* = 13), AmpC alone (34%, *n* = 12), or ESBL alone (29%, *n* = 10). *E. cloacae* was the only species that was associated with gut shedding (*p* = 0.019; Figure 1, Table 2, and Supplementary Table S1).

### Antibiotic Susceptibility Profiles of ESBL/AmpC-E

The antibiotic susceptibility profiles of the ESBL/AmpC-E isolates were diverse (Supplementary Table S1). For isolates producing both ESBLs and AmpC (*n* = 13), resistance rates were 100% to amoxicillin/clavulanate, 38% to fosfomyicin, and 31% to nitrofurantoin (intermediate susceptibility). All isolates were susceptible to carbapenems, gentamicin, and trimethoprim/sulfamethoxazole. For AmpC-E (*n* = 12), resistance rates were 100% to amoxicillin/clavulanate, 33% to fosfomyicin, 17% to ofloxacin, 8% to ciprofloxacin, and 25% to nitrofurantoin (intermediate susceptibility). All AmpC-E isolates were susceptible to gentamicin and trimethoprim/sulfamethoxazole. For ESBL-E (*n* = 10), resistance rates were 50% to trimethoprim/sulfamethoxazole, 20% to ofloxacin, 33% to ciprofloxacin, and 10% to gentamicin. All ESBL-E isolates were susceptible to amoxicillin/clavulanate (one isolate had an intermediate susceptibility), piperacillin/tazobactam, fosfomyicin, and nitrofurantoin (Supplementary Table S1).

### Genotyping of the ESBL/AmpC-E Isolates and Resistance Genes

To understand the genetic relatedness between ESBL/AmpC-E strains within and between different petting zoos, we performed ERIC PCR, followed by MLST analysis. We performed ERIC PCR on 30 isolates that were kept and stored successfully. *E. cloacae* revealed 14 isolates with 13 different clusters, *E. coli* revealed 11 isolates with six different clusters and *Citrobacter freundii* revealed five isolates with three different clusters (Supplementary Figure S1). MLST analysis performed on *E. cloacae* complex demonstrates that isolates belonged to multiple STs, of which seven are known STs, four (ST1151–ST1154) were assigned as new STs possessing new allele combinations, and one (ST1189) was assigned as a new ST possessing five new alleles: dnaA-329, fusA-215, gyrB-356, leuS-402, and rplB-153 (Figure 1).

*Escherichia coli* was the second most prevalent β-lactamase-producing species (31%, *n* = 11/35), in which the majority of the isolates (90%, *n* = 10/11) were ESBL-producers and only one strain (ST224) was an AmpC-producer carrying a *bla*_CMY–__2_. The ESBL genes detected in this species belonged to either the *bla*_CTX–M–__1_ group (three isolates: *bla*_CTX–M–__28_), the *bla*_CTX–M–__9_ group (one isolate: *bla*_CTX–M–__14_), and the *bla*_SHV_ group (*bla*_SHV–__12_, *bla*_SHV–__31_, *bla*_SHV–__2_, and *bla*_SHV–__2__a_, each detected in a different, single isolate). The 11 ESBL-producing *E. coli* isolates belonged to six known STs, including ST656 (four ring-tailed coatis from petting zoo #7), ST648 (two meerkats from petting zoo #6), ST127 (two ferrets from petting zoo #8), and three single-isolate STs: ST4981, ST2521, and ST224 (Figure 1). Neither of the *E. coli* isolates belonged to the worldwide ESBL-producing *E. coli* ST131 lineage.

The third species recovered from animals was the AmpC-producer *C. freundii* (five isolates; Figure 1 and Supplementary Table S1). Three isolates carried the *bla*_CMY–__2_ gene; two of these isolates were also ESBL-producers, one produced *bla*_CTX–M–__28_, and the other ESBL gene was not identified. Genotyping revealed the presence of two different strains shed by different animal species in zoo #6 (ST124 and ST367), and one *C. freundii* strain, assigned with a new ST, ST479, encoding two new alleles: aspC-177 and dnaG-167, shed by three individual coatis housed together in petting zoo #7 (Figure 1), suggesting inter-animal spread.

### Risk Factor Analysis for ESBL/AmpC-E Shedding

In a Univariable analysis, the shedding of an ESBL-E or an AmpC-E or ESBL/AmpC-E by an individual animal was found to be significantly associated with antibiotic treatment (*p* = 0.038, *p* = 0.011, and *p* = 0.029, respectively; Table 3). Overall, 11% of the sampled animals (*n* = 25/228) were treated with antibiotics, including trimethoprim/sulfamethoxazole, cephalosporins, doxycycline, metronidazole, chloramphenicol, and the veterinarian quinolones enrofloxacin and marbofloxacin. ESBL/AmpC-E shedding was not associated with any specific antibiotic agent. AmpC-E and ESBL-E shedding were associated with antibiotic treatment, the permitted petting policy, and the petting zoo sampled (Table 3). These factors were included in a logistic regression model, which revealed that antibiotic therapy is a risk factor for ESBL/AmpC-E shedding (OR = 7.34, 95% CI 1.88–28.56). In addition, petting zoo #2 was found to be a protective factor against ESBL/AmpC-E carriage (OR = 0.078, 95% 0.007–0.92).

**TABLE 3:** Associations and risk factor analysis for ESBL-E/AmpC shedding.

**Risk factor/Sampling site**	**AmpC shedding**			**ESBL shedding**			**Overall resistance shedding**	**Logistic regression OR**
	**Gut**	**Skin/fur/feathers**	**Total**	**Gut**	**Skin/fur/feathers**	**Total**		
Antibiotic treatment^a^	0.012^e^	0.128	0.011^e^	0.069	0.457	0.038^e^	0.029^e^	7.34
Permitted petting policy	0.002^e^	0.6	0.015^e^	0.012^e^	0.58	0.057	0.021^e^	Not included
Petting zoo	0.186	0.191	0.017^e^	0.073	0.073	0.039^e^	0.077	0.078^b^
Animal class^c^	0.255	0.024^e^	0.055	0.25	1	0.269	0.215	Not included^f^
Animal diet^d^	0.802	0.251	0.5	0.629	0.159	0.26	0.302	Not included^f^
Animal gender	0.914	0.351	1	0.641	1	0.927	1	Not included^f^

*^*a*^Any antibiotic treatment given to a specific animal during the past year. ^*b*^Petting zoo #2 was found as a protective factor. ^*c*^Mammal/reptile/avian species. ^*d*^Diet as described by the owner and categorized as various plant materials (hay, vegetables, or concentrated vegetative feed), insects, or other prey (mice). ^*e*^*p* < 0.05. ^*f*^Not included: excluded from the logistic regression model due to *p* > 0.2.*

## Discussion

This prospective study investigated the shedding of ESBL/AmpC-producing Enterobacteriaceae in a large and highly diverse sample of petting zoo animal species. To the best of our knowledge, this is the first study that focuses specifically on ESBL/AmpC-E shedding and defines the related risk factors. Of the 228 animals sampled throughout the country, 12% shed ESBL/AmpC-E. The prevalence of animal shedding varied significantly between different petting zoos, which may be explained by the diverse facilities that were sampled and that represent various animal–visitor interfaces. Because data regarding the prevalence of ESBL/AmpC-E in petting zoos in other countries are unavailable, these data are incomparable with other studies.

Although ESBL/AmpC-E shedding was previously reported in various mammals, reptiles, and avian species (Vittecoq et al., 2016), we screened a highly diverse population of new animal species. We report, for the first time, ESBL/AmpC-E shedding in 13 new host species of mammals, reptiles, and avian species (Figure 1). We found that ESBL/AmpC-E gut shedding was independent of the type of animal species, possibly due to the small number of individual animals sampled within each species. The study included both fecal and surface sampling of diverse animal species, from smooth-skin reptiles to feathered birds or large furred animals, such as sheep or deer. Therefore, ESBL/AmpC-E recovery could be influenced by the type and area of the sampled surface. Supporting this claim is the finding that, in seven animals from four petting zoos, we recovered two different ESBL/AmpC-E strains from the feces and body surface of the same animal, indicating that ESBL/AmpC-E surface shedding may be due to fecal or environmental contamination, rather than gut shedding. In spite of these obstacles, the presence of MDR bacteria on animal surfaces highlights the potential risk of ESBL/AmpC transmission from healthy shedding animals to children and other visitors due to the possible close contact through petting and animal holding.

Importantly, all 35 ESBL/AmpC-E isolates recovered from animals belonged to only three different genera – *Enterobacter cloacae*, *E. coli*, and *C. freundii* – with *E. cloacae* being the most prevalent species, and which was found to be significantly associated with gut shedding. *Enterobacter* was previously reported to be a commensal bacteria shed by animals in zoos (Ahmed et al., 2007) and a pathogen causing infections in animals (Gibson et al., 2010), but it is less frequently reported as a major ESBL/AmpC-producing genus in animals. The main investigated and reported ESBL species in the literature is *E. coli.* However, in this study we sampled a variety of different species, representing a variety of environmental conditions and interfaces. *E. cloacae* is ubiquitous in terrestrial and aquatic environments, such as water, sewage, soil, and food (Davin-Regli and Pagès, 2015). We hypothesize that due to the heterogeneous study population, we detected a high prevalence of *E. cloacae*. Previous reports on zoonotic bacterial outbreaks in petting zoos focused on highly transmissible virulent pathogens, such as *E. coli* O157:H7 (Stirling et al., 2007; Conrad et al., 2016), *Salmonella* spp., *Campylobacter* (Bender and Shulman, 2004), and *Shigella* (Stirling et al., 2007). Although *Enterobacter* and *Citrobacter* are known to be AmpC-producing human pathogens (Santos et al., 2015), they were not described previously as potential zoonotic bacteria. In light of our findings, these genera should be recognized as a possible source for ESBL/AmpC and it is recommended that they be actively monitored in petting zoo facilities.

Bacterial genotyping revealed multiple sequence types, which varied both between and within the same facility. Alarmingly, among the different ESBL-producing *E. coli* STs recovered, we identified three distinct pathogenic *E. coli* strains: ETEC ST656 (Oh et al., 2014) and UPEC ST127 (Gibreel et al., 2012), which have both been described as highly virulent, and ST648, which has previously been reported in humans and in domestic and wild animals (Ewers et al., 2014). Each of these three highly virulent *E. coli* STs was recovered from the same mammalian species, housed in the same cage, demonstrating inter-animal clonal transmission that could be explained by animal-to-animal contact or by environmental shedding. These strains were found in two petting zoos (#7 and #8) that had a permitted petting policy; thus, transmission may be a relevant risk and could be mediated, e.g., via workers and environmental shedding. In addition, we identified five new *E. cloacae* STs and one new *C. freundii* ST, which may suggest that these are commensal/environmental strains that may have acquired the ESBL/AmpC resistance via plasmid or gene acquisition. The high diversity of bacterial STs and resistance genes indicates the complex transmission mechanisms and the possible involvement of horizontal transfer of ESBL/AmpC genes and plasmids among petting zoo animals.

We found antibiotic treatment to be a risk factor for ESBL/AmpC-E shedding, similar to previous data on ESBL shedding in both animals (Belas et al., 2014) and humans (Ben-Ami et al., 2009). Assuming that human–animal contact is a risk factor for bacterial transmission in petting zoos, antibiotic-treated animals may constitute a high-risk population for resistant bacterial transmission. In light of these findings, it may be beneficial to consider the interactions between antibiotic-treated animals and visitors. In addition, we found that such animals are treated with a variety of antimicrobials, including third-generation cephalosporins and quinolones. An appropriate guideline for antimicrobial use in petting zoos is currently lacking, and our findings call for the establishment of such a guideline. A recommendation for isolation of antibiotic-treated animals should further studied, since the duration of ESBL/AmpC shedding in animals was not established.

Another important finding was that ESBL/AmpC-E shedding was significantly associated with the petting permission policy. Strong support for this correlation resides in our findings that petting zoo #2, which had a petting prohibition policy, was found to be a protective factor for ESBL/AmpC-E carriage. We also found ESBL/AmpC-E carriage on the surfaces of animals (skin, fur, or feathers) – an exceptionally important finding in petting zoos, where direct contact is the main interaction between visitors (mostly children) and animals. These findings emphasize the connection between human–animal contact and ESBL/AmpC-E shedding, and they further highlight the importance of implementing strict hygiene and prevention guidelines.

In summary, the data reported in the current study raise new concerns regarding petting zoos as possible sources for ESBL/AmpC-E due to environmental shedding. Considering the valuable contribution of animal-associated activities to physical, social, and psychological aspects of human health (Friedmann et al., 2015), the educational and psychological importance of petting zoos is unequivocal. Therefore, we highly recommend promoting appropriate guidelines and interventions, some of which may include immediate actions and should be further investigated and implemented in the future. Immediate recommendations for petting zoo owners constitute the implementation of hygiene guidelines, including accessible means for hand washing and disinfection, as well as restricted refreshment areas for visitors. Long-term recommendations may comprise improved antibiotic stewardship and the implementation of active surveillance programs. Our findings emphasize the need of additional national and international surveillance studies, which, together, should facilitate the establishment of a standard comprehensive guideline for petting zoo operators and visitors, so as to minimize the associated public health risks.

## Data Availability Statement

The datasets generated for this study can be found in the https://pubmlst.org/. Accession numbers: ST1151–ST1154 and ST1189.

## Ethics Statement

The animal study was reviewed and approved by the Internal Ethics Committee of the Koret School of Veterinary Medicine, Israel (Protocol KSVM-VTH/25_2016).

## Author Contributions

AS-T and GM collected and analyzed the specimens and petting zoo owners’ questioners. AS-T performed the molecular analysis. YP performed the bacterial identification. AS-T and WA performed the statistical analysis. AS-T, AS, and SN-V contributed to the conception and design of the study. AS and SN-V wrote the manuscript. All authors read and approved the submitted manuscript.

## Conflict of Interest

The authors declare that the research was conducted in the absence of any commercial or financial relationships that could be construed as a potential conflict of interest.

## Footnotes

1 https://www.cdc.gov/features/animalexhibits/index.html

2 https://pubmlst.org/
