# Supplementary material for: Petting Zoo Animals as an Emerging Reservoir of Extended-Spectrum β-Lactamase and AmpC-Producing Enterobacteriaceae
Source: Front Microbiol. 2019 Oct 30;10:2488. doi: 10.3389/fmicb.2019.02488 (PMC6831544; doi:10.3389/fmicb.2019.02488)
Supplement: Supplementary file 1 [file Data_Sheet_1.pdf]

Supplementary Data

Table S1. Characteristics, origin, and antibiograms of 35 ESBL/AmpC-producing *Enterobacteriaceae* recovered from petting zoo animals

Zoo	Isolate No.	ID	Host animal	Class[a]	Sample origin	Resistance Phenotype	Antibiogram[b] AMC	TZP	LEX	CXM	FOX	CAZ	CRO	CTX	IPM	ETP	MEM	CIP	OFX	AMK	GEN	FOF	NIT	SXT
2	P33.1	*E. cloacae*	*Capra nubiana* [c]	M	feces	AmpC	■	▒	■	■	■		■	■					■					
4	P57.1	*E. cloacae*	*Mesocricetus auratus*	M	feces	AmpC	■		■	■	■		■									■		
	P57.2	*E. cloacae*	*Mesocricetus auratus*	M	feces	ESBL+ AmpC	■	■	■	■	■	■	■	■								■		
	P62.1	*E. cloacae*	*Pyrrhura molinae* [c]	A	feathers	ESBL+ AmpC	■	■	■	■	■	■	■	■								■		
	P67.1	*E. cloacae*	*Cavia porcellu* [c]	M	skin/fur	ESBL+ AmpC	■	■	■	■	■	■	■	■									▒	
	P73.1	*E. cloacae*	*Gerbillus* [c]	M	skin/fur	ESBL+ AmpC	■	■	■	■	■	■	■	■									▒	
	P82.2	*E. cloacae*	*Oryctolagus cuniculus*	M	feces	AmpC	■		■	■	■		■	▒										
5	p265.2	*E. cloacae*	*Oryctolagus cuniculus*	M	feces	ESBL+ AmpC	■	■	■	■	■	■	■	■								■		
	p273.2	*E. coli*	*Oryctolagus cuniculus*	M	feces	ESBL			■	■		■	■	■				■						
6	P296.1	*C. freundii*	*Eublepharis macularius* [c]	R	feces	ESBL+ AmpC	■		■	■	■	■	■	■										
	P300.2	*E. cloacae*	*Correlophus ciliates* [c]	R	feces	ESBL+ AmpC	■		■	■	■		■	▒									▒	
	P304.2	*E. cloacae*	*Pogona* [c]	R	skin	ESBL+ AmpC	■	■	■	■	■	■	■										▒	
	P307.2	*E. cloacae*	*Pogona* [c]	R	skin	AmpC	■		■	■	■		■										▒	
	P312.1	*E. cloacae*	*Pantherophis guttatus* [c]	R	feces	AmpC	■		■	■	■		■									■		
	P312.2	*E. cloacae*	*Pantherophis guttatus* [c]	R	feces	ESBL+ AmpC	■		■	■	■		■	▒								■		
	P323.1	*E. cloacae*	*Python regius*	R	feces	ESBL+ AmpC	■	■	■	■	■	■	■	■										
	p360.1	*E. coli*	*Capra aegagrus hircus*	M	feces	ESBL			■	■		■	■	■										
	P371.1	*E. coli*	*Suricata suricatta* [c]	M	feces	AmpC	■	▒	■	■	■	■	■	■				■	■					
	P379.3	*C. freundii*	*Suricata suricatta* [c]	M	feces	ESBL+ AmpC	■		■	■	■		■	■										
	P381.2	*E. coli*	*Suricata suricatta* [c]	M	feces	ESBL			■	■	▒	■	■	■				■	■					■
	P382.2	*E. coli*	*Suricata suricatta* [c]	M	skin/fur	ESBL	▒		■	■	■	■	■	■				■	■		■			
7	P105.1	*E. coli*	*Nasua nasua* [c]	M	feces	ESBL			■	■		■	■	■										■
	P105.2	*C. freundii*	*Nasua nasua* [c]	M	feces	AmpC	■	■	■	■	■	■	■	■										
	P106.1	*E. coli*	*Nasua nasua* [c]	M	feces	ESBL			■	■		■	■	■										■
	P106.2	*C. freundii*	*Nasua nasua* [c]	M	feces	AmpC	■	■	■	■	■	■	■	■										
	P107.1	*E. coli*	*Nasua nasua* [c]	M	feces	ESBL			■	■		■	■	■										■
	P108.1	*E. coli*	*Nasua nasua* [c]	M	feces	ESBL			■	■		■	■	■										■
	P108.2	*C. freundii*	*Nasua nasua* [c]	M	feces	AmpC	■	■	■	■	■	■	■	■										
	P114.1	*E. cloacae*	*Testudo graeca* [c]	R	skin	AmpC	■		■	■	■		■	■								■		
8	P151.2	*E. cloacae*	*Centrochelys sulcate* [c]	R	skin	AmpC	■		■	■	■		■	■								■	▒	
	P152.2	*E. cloacae*	*Centrochelys sulcate* [c]	R	feces	ESBL+ AmpC	■	■	■	■	■	■	■	■										
	P203.1	*E. coli*	*Mustela putorius furo* [c]	M	feces	ESBL			■	■		■	■	■										
	P205.1	*E. coli*	*Mustela putorius furo* [c]	M	feces	ESBL			■	■		■	■	■										
	P208.1	*E. cloacae*	*Pogona* [c]	R	feces	ESBL+ AmpC	■	■	■	■	■	■	■	■								■		
	P215.1	*E. cloacae*	*Mus*	M	feces	AmpC	■		■	■	■		■										▒	

[a] A, avian; M, mammal; R, reptile.

[b] Determined by Vitek-2. The color of the squares represents the susceptibility to the indicated drug; Black: resistance; Grey: intermediate resistance; and White: sensitive. Acronyms: AMC: amoxicillin-clavulanate, TZP: tazobactam-piperacillin, LEX: cephalexin, CXM: cefuroxime, FOX: cefoxitin, CAZ: ceftazidime, CRO: ceftriaxone, IPM: imipenem, ETP: ertapenem, MEM: meropenem, CIP: ciprofloxacin, OFL: ofloxacin, AMK: amikacin, GENT: gentamicin, FOF- fosfomicin, NIT- nitrofurantoin, SXT- trimethoprim-sulfamethoxazole.

[c] Animal species described here for the first time as shedding ESBL/AmpC-E

Figure S1

Enterobacterial repetitive intergenic consensus (ERIC) PCR on 30 ESBL/AmpC-producing isolates. These isolates belonged to *Enterobacter cloacae* (n=14, 13 clusters); *E. coli* (n=11, six clusters) and *Citrobacter freundii* (n=5, three clusters). Isolates that exhibited an identical ERIC PCR pattern were classified as belonging to the same cluster. MLST was performed on one isolate from each cluster. Isolates for which MLST was performed are designated in bold and sequence types (ST) are marked at the bottom of each ERIC PCR analysis.

Enterobacter cloacae

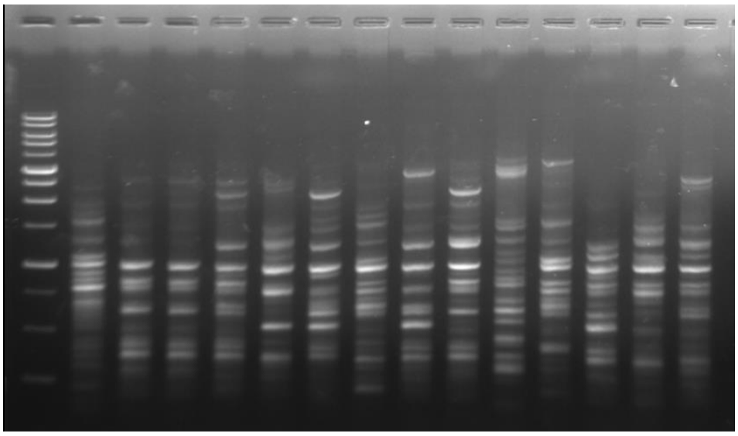

ST **1151** **750** 750 **350** **557** **170** **102** **1152** **1153** **1189** **1154** **112** **182** **511**

E. coli

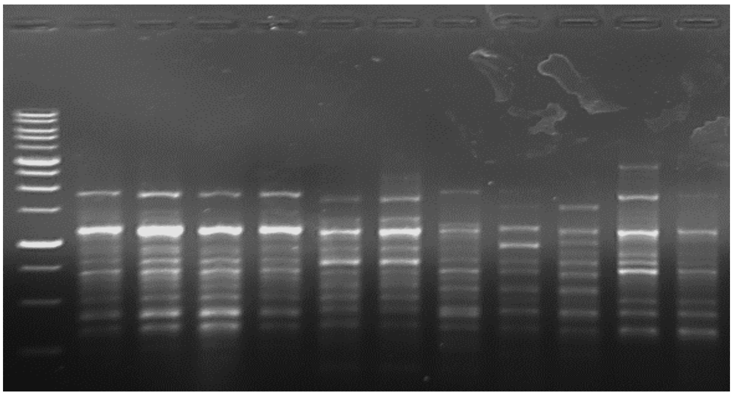

ST **656** 656 656 656 **127** 127 **4981** **2521** **224** **648** **648**

Citrobacter freundii

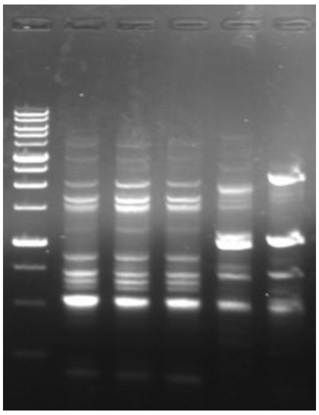

ST **479** 479 479 **124** **367**
